# The Impact of the Internet on Health Consultation Market Concentration: An Econometric Analysis of Secondary Data

**DOI:** 10.2196/jmir.6423

**Published:** 2016-10-28

**Authors:** Jia Li, Ya Zhang, Ling Ma, Xuan Liu

**Affiliations:** ^1^ E-commerce Institute School of Business East China University of Science and Technology Shanghai China

**Keywords:** long tail effect, superstar effect, E-consultation, market concentration, information asymmetry, signaling theory, online reputation, self-representation

## Abstract

**Background:**

Many markets have traditionally been dominated by a few best-selling products, and this is also the case for the health care industry. However, we do not know whether the market will be more or less concentrated when health care services are delivered online (known as E-consultation), nor do we know how to reduce the concentration of the E-consultation market.

**Objective:**

The aim of this study was to investigate the concentration of the E-consultation market and how to reduce its concentration through information disclosure mechanisms (online reputation and self-representation).

**Methods:**

We employed a secondary data econometric analysis using transaction data obtained from an E-consultation Website (haodf.com) for three diseases (infantile pneumonia, diabetes, and pancreatic cancer) from 2008 to 2015. We included 2439 doctors in the analysis.

**Results:**

The E-consultation market largely follows the 20/80 principle, namely that approximately 80% of orders are fulfilled by nearly 20% of doctors. This is much higher than the offline health care market. Meanwhile, the market served by doctors with strong online reputations (beta=0.207, *P*<.001) or strong online self-representation (beta=0.386, *P*<.001) is less concentrated.

**Conclusions:**

When health care services are delivered online, the market will be more concentrated (known as the “Superstar” effect), indicating poor service efficiency for society as a whole. To reduce market concentration, E-consultation websites should provide important design elements such as ratings of doctors (user feedback), articles contributed by doctors, and free consultation services (online representation). A possible and important way to reduce the market concentration of the E-consultation market is to accumulate enough highly rated or highly self-represented doctors.

## Introduction

### Background

The Pareto principle (also known as the 80/20 rule) states that, in many cases, approximately 80% of the effects result from 20% of the causes. The Pareto principle is very popular in the economic market, as it indicates that a small proportion (eg, 20%) of products in a market often generate a large proportion (eg, 80%) of sales [1]. For example, a relatively small number of titles by established best-selling authors account for a high percentage of book sales, Billboard’s “top 40” hits account for the majority of radio playlists and music sales, and movie rentals are dominated by a few popular “new releases.”

Not surprisingly, the Pareto principle also applies to the health care service market. In the health care service market, a few of the best hospitals or doctors have a much higher market share than do ordinary hospitals or doctors [2]. Market concentration or the Pareto principle may be an advantage in a business context (eg, in a supermarket or bookstore), but it is not good for the health care industry. A major concern for the health care industry is the limited service capability of each hospital or doctor; this is, of course, not a problem in the product sales context. When the market is highly concentrated on a few very good doctors, the efficiency of health service delivery to society as a whole will be negatively affected. That is, a few very good doctors will be extremely busy, while some other unknown doctors will be idle [3]. Therefore, a low concentrated market with balanced supply and demand is the ideal condition for the health care industry. Many efforts have been made to decrease the concentration of the health care market by balancing supply and demand. One example of such efforts is the role of family physicians in western countries, who make up a sizable portion of the primary care workforce [4]. A patient must see a family physician before seeing doctors at higher-level hospitals. Another example is the hierarchical diagnosis and treatment system in China, an important part of China’s medical reform [5]. To encourage patients to see doctors initially at local, lower-level hospitals, China implemented new policies such as charging less at lower-level hospitals and increasing the rates that medical insurers pay to these hospitals.

A recent trend in eHealth is delivering health care services online [6,7]. Among online health services, E-consultation seems to be particularly attractive and is increasing in popularity [8-10]. This new type of online health care consultation can reduce both waiting time and travel expenses. It is also likely to be a valuable option in terms of providing patients with more efficient diagnoses. Most important, patients who have limited or even no medical resources have equal access to medical experts online, leading to better and more efficient use of nationwide medical resources. Online health care consultation will be of great significance in reducing medical costs and in improving the operational efficiency, effectiveness, and equity of medical resources, as well as in enhancing customer satisfaction. Therefore, the use of E-consultation is becoming popular and has been increasing rapidly in recent years [11]. It still has some downsides such as overreliance on it, which can lead to delays in care delivery, patients’ privacy and confidentiality, and technical difficulties involving such technology. Examples of third party E-consultation websites include askthedoctor.com [12], askdoctorfree.com [13], and haodf.com [14]. E-consultation has also been used by some offline medical institutions, such as the Mayo Clinic, to deliver health care services online [15-17]. E-consultation appears to have improved access to specialists, who can be integrated into care processes when timely expert opinions are needed.

The new technologies embedded in E-consultation are not limited to digital communications, computing, and storage but also involve a qualitative transformation in search tools, recommendation tools, and social network technologies [18]. Therefore, E-consultation not only provides convenience and better decision results for the user but also changes the costs of seeking information and patterns of searching for information. Because the choice of a doctor is made based on the information received, the concentration of the online market will be different from the offline market. Two possible consequences of Internet technology on these concentrations have been observed in the E-commerce market, namely the “Long-Tail” effect and the “Superstar” effect. On one hand, the user can find more niche (unpopular) doctors at much lower searching costs than ever before, creating a “Long Tail” in the concentration of demand for doctors. On the other hand, users can more easily find the most high-profile doctors online, creating “Superstar” or “winner-take-all” markets where some very good doctors dominate the market [19]. However, we have no idea whether the E-consultation market will be more of a superstar market or a long-tail market compared with a traditional offline context. What can website designers do to turn the superstar market into a long-tail market?

In this study, we aim to investigate the following research questions:

RQ1: Will E-consultation be more of a long-tail or a superstar market? Or, will the E-consultation market be less concentrated or more concentrated than the offline market?

RQ2: Can information disclosure mechanisms (the doctor’s online reputation and self-representation) help reduce market concentration?

### Research Hypotheses

#### Star Effect Versus Long-Tail Effect

Choosing a doctor on a website is totally different from choosing a doctor at an offline hospital. A significant difference is the information available to the user when making a decision. With the help of information technology such as search engines, recommendation tools, and social networking technologies, the user can easily reach more doctors (especially unknown doctors) at a much lower cost than before. In the traditional offline context, the user’s choice set of doctors is quite small. The user usually chooses a doctor near their home or workplace. However, in the online context, the user can choose any doctor nationwide with just a few clicks of the mouse. This means that the choice set in the context of E-consultation is much larger, and users have more of an opportunity to choose unknown doctors than ever before. Thus, the online market will be less concentrated on a small number of high-profile doctors, creating a long-tail effect.

Another possible consequence of E-consultation is the superstar effect, also known as the Matthew Effect or “the rich get richer.” This is because popular doctors enjoy greater visibility on E-consultation platforms (eg, they are ranked highly by search engines or recommended preferentially by websites). As a consequence, the very good and popular doctors have a greater chance than before of being identified at the national level, which further increases their chance of being chosen by users. Thus, the online market will be more concentrated on a small number of famous doctors, creating a superstar effect.

In summary, both the long-tail and superstar effects may exist in the E-consultation context. We cannot know which effect will be dominant without an empirical study. Therefore, we propose the following two competitive hypotheses:

H1a: The online market is less concentrated than the offline market.

H1b: The online market is more concentrated than the offline market.

#### Information Asymmetry Theory and Information Disclosure

Health care is a market with high information asymmetry. Information asymmetry models assume that at least one party to a transaction has relevant information while the others do not. In the case of E-consultation, doctors have more information about their own service quality than do the patients. Although doctors know their own service quality, patients have little information on this very important question. This situation of information asymmetry creates an imbalance of power in transactions, which can sometimes cause the transactions to go awry—a type of market failure in a worst-case scenario.

According to signaling theory [20], information asymmetry can be reduced by one party (termed the agent) credibly conveying information about itself to another party (the principal). The recipient of the information can interpret the received signal and adjust their purchases accordingly. For example, in Spence’s job-market signaling model [20], (potential) employees send signals about their abilities to employers by acquiring education credentials. The informational value of the credential comes from the fact that the employer believes the credential is positively correlated with having greater ability and is difficult for less-able employees to obtain. Thus, the credential enables the employer to reliably distinguish low-ability workers from high-ability workers.

In the E-consultation context, doctors send information about their service quality to patients. After receiving this information, patients may change their judgment about doctors’ service quality and further change their choice of doctors. In this study, we focus on two signals that a doctor can send about their service quality on an E-consultation website, specifically, online reputation and online self-representation.

#### Doctors’ Online Reputations

An online reputation (also known as online word-of-mouth) is built based on feedback from patients. E-consultation websites usually provide a feature known as “rate a doctor.” A patient who has visited the doctor previously can write a review of the doctor in terms of technical competence, interpersonal manner, systems issues, etc. The online reputation system is very popular on E-commerce platforms and has been demonstrated as a reliable mechanism to reduce market information asymmetry. For example, eBay uses a system of customer feedback to publicly rate each member. Amazon [21] has a similar reputation mechanism in place, and merchants develop their reputations across different dimensions [22]. According to a recent study, a doctor’s online reputation, as rated by patients is a good indicator of that doctor’s service quality in the real world [7]^.^

If the E-consultation website does not provide an online reputation feature, the user judges the doctor’s service quality based only on the doctor’s professional standing (eg, director, associate director). Therefore, the user’s consideration set is small because only those doctors with high offline positions will be considered. When the E-consultation website does provide an online reputation feature, users have more clues to evaluate the doctor’s service quality. If the market has many doctors with strong reputations, users will consider those who are highly rated but perhaps have lower offline positions. This means that the consideration set is enlarged. However, if the market is full of doctors with poor reputations, users will not include those poorly rated doctors in the consideration set. This means the consideration set remains at the same size or is even smaller (if doctors with high positions are poorly rated). Thus, having a market with highly rated doctors is very important. If the market has many highly rated doctors, market efficiency will be improved because users have more credible doctors from which to choose (ie, the supply of high-quality doctors is increased). In the same vein, market efficiency will not be improved if the market has few highly rated doctors. Therefore, we propose the following hypothesis:

H2: A market served by many doctors with strong online reputations is less concentrated than a market served by many doctors with poor online reputations.

#### Doctors’ Online Self-Representation

Self-representation is the activity a doctor commits online for the purpose of sending quality information. There are several ways for doctors to represent themselves on an E-consultation website. For example, a doctor can post articles or provide free consultation services. Such efforts are another type of signal the doctor sends to users. The user can evaluate the doctor’s service quality in terms of the efforts reflected online. For example, doctors who post popular medical science articles demonstrate not only their medical knowledge and skills but also their positive attitudes toward E-consultation as well as their Internet savvy. Meanwhile, the quality and number of free consultation services provided are good indicators of the doctor’s expertise and social responsibility.

Therefore, when an E-consultation website provides self-representation features, the users have more information with which to judge the doctor’s service quality. If the market has many doctors representing and promoting themselves, users will consider these highly represented doctors, who may not be well known offline. This means the consideration set, as well as the supply of high quality doctors, is enlarged. However, if the market is full of doctors with low self-representation, users will not consider these low-effort doctors, and thus the consideration set remains the same. For the same reason, if the market has many highly represented doctors, market efficiency will be improved because users will have more credible doctors from which to choose. Therefore, we propose the following hypothesis:

H3: A market served by many doctors who are highly represented online is less concentrated than a market served by many doctors who are not well represented online.

In summary, we aim to investigate the concentration of the E-consultation market and how to reduce its concentration through information disclosure mechanisms. We hypothesize that the online market is less or more concentrated than the offline market, and the online reputation or self-representation can be used to reduce market concentration.

## Methods

### Data Collection

We employ a secondary data analysis as the research method. Secondary data refers to data that were collected by someone other than the researcher. Primary data, by contrast, are collected by the investigator conducting the research. In this study, the data were originally collected by the E-consultation website, haodf.com [14]. We developed a network spider to crawl data from the website indirectly. Therefore, the data used in this study are secondary data.

We collected data from Good Doctor (haodf.com [14]) to test the hypotheses proposed in the previous section. Haodf.com is a leading E-consultation website in China. As of May 2016, the platform had 397,587 registered doctors from 5332 regular hospitals. Good Doctor provides two types of consultation services: free consultation using written asynchronous communication and consultation via phone for which there is a charge. Haodf.com provides a home page for each doctor, which contains their demographic information, clinic information, service fees, user feedback, contributed articles, and service records (both free and paid services). Each user can choose any doctor from the website, as long as the doctor is providing service at that moment. The search engine, the recommendation systems, and the social networks are all accessed on haodf.com. Therefore, the doctor’s online word of mouth, contributed articles, and free and paid services are all accessible to the users. A network spider was used to collect data from the site about three diseases (infantile pneumonia, diabetes, and pancreatic cancer) from 2008-2015. The three diseases were chosen because we intend to cover both acute and chronic diseases, as well as both high mortality rate and low mortality rate diseases.

### Empirical Model

Following Brynjolfsson et al’s work [1], we fit the sales, sales rank, reputation, and self-representation data to the following log-linear relationship. More details about the empirical econometric model are provided in Multimedia Appendix 1.

### Measures

Online reputation is measured by the number of votes, letters of thanks, and gifts received by the doctor (the three variables are standardized and then averaged to create a composite variable). The review score is not used to measure online reputation in this study because we observe a ceiling effect (most doctors have a top score, making it very difficult to distinguish doctors). Self-representation is measured by the number of scientific papers the doctor has contributed and the number of free services they have provided (the two variables are standardized and then averaged to create a composite variable).

Control variables include the doctor’s position, hospital level, service price, and duration of providing online service. Position is measured on a scale of 1-5, with 1 being the lowest and 5 the highest. Hospital level is measured on a scale of 1-3, with 1 being the lowest and 3 the highest. Service price is measured by the service fee (in Chinese Yuan) per phone call. Duration is measured by the number of months since the doctor’s homepage was established.

## Results

### Summary Statistics

The descriptive statistics of variables used in this study are shown in Table 1. The correlations between major variables are listed in Table 2.

**Table 1:** Descriptive statistics.

Variable	Observations	Mean	SD	Min.	Max.
Order number	2439	341.360	826.100	1	12518
Order rank	2439	461.880	322.077	1	1231
Vote	2439	21.320	34.134	1	429
Gift	2439	17.791	61.205	0	1003
Thank-you letter	2439	5.988	13.203	0	157
Reputation	2439	0.000	0.922	-0.447	13.162
Articles	2439	13.242	145.257	0	6871
Free service	2439	319.798	783.135	0	10876
Self-represent	2439	0.000	0.733	-0.250	23.536
Position	2439	4.221	1.067	1	5
Hospital level	2439	2.824	0.529	1	3
Service price	2439	147.984	44.283	0	1200
Online duration	2439	53.024	27.465	1	94

**Table 2:** Variable correlations (Pearson correlation coefficient).

	Order number	Reputation	Self-endeavor	Position	Hospital level	Service price	Online duration
Order number	1						
Reputation	.581	1					
Self-represent	.715	.428	1				
Position	.001	.090	.018	1			
Hospital level	.018	.004	.019	.062	1		
Service price	.040	.161	.012	.118	.009	1	
Online duration	.200	.290	.183	.106	-.049	.079	1

**Table 3:** Regression results.

Variable	Model 1 (standard error)	Model 2 (standard error)	Model 3 (standard error)	Model 4 (standard error)
Position	-0.082^a^ (0.043)	-0.007 (0.021)	-0.001 (0.019)	0.005 (0.018)
	*P*=.06	*P=*.73	*P*=.96	*P*=.76
Level	0.119 (0.086)	0.007 (0.041)	0.010 (0.038)	0.014 (0.035)
	*P*=.17	*P*=.87	*P*=.80	*P*=.68
Price	0.001 (0.001)	0.0003 (0.0005)	0.0004 (0.0005)	-0.0003 (0.0004)
	*P*=.20	*P*=.49	*P*=.41	*P*=.45
Duration	-0.022 (0.002)	-0.001 (0.010)	-0.0002 (0.001)	0.001 (0.001)
	*P*<.001	*P*=.43	*P*=.82	*P*=.27
Lrank		-1.950 (0.022)	-2.086 (0.024)	-2.301 (0.024)
		*P*<.001	*P*<.001	*P*<.001
Reputation			-1.011 (0.052)	
			*P*<.001	
Reputation*Lrank			0.207 (0.012)	
			*P*<.001	
Self-represent				-2.024 (0.066)
				*P*<.001
Self-represent*Lrank				0.386 (0.014)
				*P*<.001
Constant	2.439 (0.330)	15.056 (0.211)	15.840 (0.204)	17.266 (0.200)
	*P*<.001	*P*<.001	*P*<.001	*P*<.001
R^2^	0.068	0.786	0.815	0.845

^a^*P*<.1.

### Evaluation Outcomes

The regression results are shown in Table 3. Model 1 contains only control variables, which builds a benchmark for the following models.

**Figure 1:**
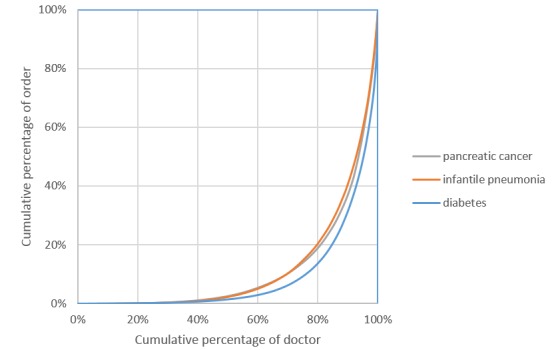
Lorenz curve.

Model 2 includes the order rank. The results from Model 2 show that the order rank is negatively related to the number of orders (beta_1_ =-1.950, *P*<.001). The Lorenz curve (the relationship between the cumulative percentage of doctors and the cumulative percentage of orders) for the E-consultation market is shown in Figure 1. The Lorenz curve is a graphical representation of the distribution of orders. It shows for the bottom x% of doctors, what percentage (y%) of the total order they have. The percentage of doctors is plotted on the x-axis, and the percentage of order on the y-axis. We can estimate from Figure 1 that the market concentration largely follows the 80/20 principle, such that approximately 80% of the orders are dominated by nearly 20% of doctors. The concentration of 80/20 at the doctor level is much higher than any of the offline markets [23,24]. Thus, the online market is more of a superstar market than a long-tail market. Therefore, H1a is rejected and H1b is supported.

**Figure 2:**
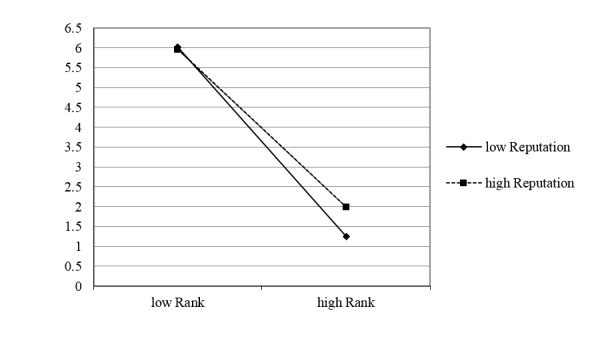
The interaction between online reputation and order rank.

Model 3 focuses on the interaction between reputation and order rank. The results of Model 3 reveal a significant negative interaction between online reputation and rank (beta_4_ =0.207, *P*<.001). A positive and significant beta_4_ (given beta_1_ is negative and significant) indicates online reputation weakens market concentration. The interaction plot between online reputation and rank is shown in Figure 2. We can see that the line for the high reputation doctors is smoother than the low reputation doctors, indicating less rank effect. This means that the market served by doctors with strong online reputations is less concentrated than the market served by doctors with low online reputations. Therefore, H2 is supported.

**Figure 3:**
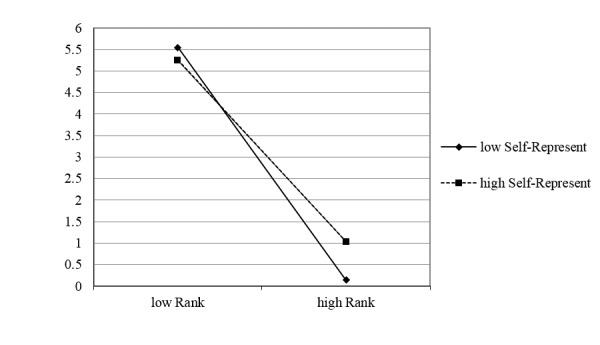
The interaction between online self-representation and order rank.

Model 4 focuses on the interaction between self-representation and order rank. The results of Model 4 reveal a significant negative interaction between online self-representation and rank (beta_5_ =0.386, *P*<.001). A positive and significant beta_5_ (given beta_1_ is negative and significant) indicates that self-representation weakens market concentration. The interaction plot between online reputation and rank is shown in Figure 3. The line for the high self-representation doctors appears smoother than the low self-representation doctors, indicating less rank effect. This means that the market served by doctors with strong online self-representation is less concentrated than the market served by doctors with low online self-representation. Therefore, H3 is supported.

### Robustness Check

We ran a robustness check by using alternative measures for reputation and self-endeavor and got similar results (see Multimedia Appendix 2).

## Discussion

### Principal Results

In this study, we found the E-consultation market to be more concentrated than the offline health care market, and both online reputation and self-representation help reduce market concentration. Specifically, we found the following. First, the E-consultation market is more concentrated than the offline health care market. In other words, the E-consultation market is more of a superstar market than a long-tail one.

Second, the market served by many doctors with strong online reputations is less concentrated than the market served by many doctors with poor online reputations.

Third, the market served by many doctors with high levels of online self-representation is less concentrated than the market served by many doctors with low levels of online self-representation.

### Comparison With Prior Work

#### The Impact of the Internet on Market Concentration

Many prior studies have investigated the effect of the Internet on sales concentration. One of the most frequently cited phenomena is the long-tail effect (ie, the online market is less concentrated than the offline one). The main drivers of the long-tail effect come from both the supply side and the demand side [25].On the supply side, as e-tailers expand, centralized warehousing allows for more offerings, thus making it possible for them to cater to more varied tastes. On the demand side, tools such as search engines, recommender software and sampling tools allow customers to find products outside of their geographic areas. The long-tail effect has been confirmed by many previous studies [1,26]. Subsequent research further distinguished the two drivers and confirmed that a significant amount of niche product consumption online is due to the direct influence of the channel on consumer behavior, not just due to selection effects from the types of consumers who decide to use an Internet channel or the types of products that consumers decide to purchase online [27]. Specifically, consumers’ use of Internet search and discovery tools, such as recommendation engines, is associated with an increase in the share of niche products [1,28,29].

Another frequently cited phenomenon is the superstar effect (ie, the online market is more concentrated than the offline one). The superstar phenomenon emerges when a comparatively small number of participants excel, surpass others in their field, and reap much greater rewards [30]. This phenomenon has been observed in virtually all categories of human activity, for example, in sports [31], music [32], entertainment [33], word frequency [34], and science [35]. Many studies observe the superstar effect when consumers move from brick-and-mortar to Internet markets [36-38]. For example, Hendricks and Sorensen [37] found that in the online music market, the distribution of sales is substantially more skewed than it would be if consumers were more fully informed. Zhong and Michahelles [38] verified that Google Play is more of a superstar market—strongly dominated by popular hit products—than a long-tail market.

Previous studies of the impact of the Internet on market concentration mainly focus on the business context. We do not know of any studies investigating the impact of the Internet on health care market concentration. The results of this study show that the E-consultation market will be more of a superstar market than a long-tail market, revealing a “rich-getting-richer” picture. Some actions (eg, providing user feedback, allowing doctors self-representation, the adoption of human or automated medical guidance) must be taken to reduce this undesirable outcome.

#### The Market Concentration in Health Care

There are previous studies on the concentration of the health care market. The most frequently investigated topic is the impact of market concentration on service prices. Previous studies reveal that higher market concentration usually leads to higher service prices [23,39,40]. For example, Dunn and Shapiro [39] found that physicians in more concentrated markets charge higher service prices; a physician in the 90th percentile of market concentration will charge 14-30% higher fees than a physician in the 10th percentile. Their estimates imply that physician consolidation has caused an approximately 8% increase in fees, on average, over the last 20 years and substantially higher increases in concentrated markets. Austin and Baker [40] found that counties with the highest average physician concentrations had prices 8-26% higher than prices in the lowest counties and concluded that physician competition is frequently associated with higher prices. However, market concentration also provides some benefits. Dunn and Shapiro [41] reveal that physician concentration has a small but statistically significant effect on service utilization. An increase in 1 standard deviation in cardiologist concentration causes a 5% increase in cardiologist service provision. Higher concentration also leads to fewer readmissions, implying potential health benefits.

Existing studies on health care market concentration are mainly conducted at the hospital level. The major reason is that most data are available at the hospital level.

E-consultation websites and historical transaction data provide a good opportunity to study market concentration at the level of individual doctors. Therefore, an important contribution of this study compared to previous studies is the unit of analysis. In addition, most previous studies are interested in the consequences of market concentration. However, we are interested in how to build a more or less concentrated health care market.

### Theoretical Contributions

Our research offers several important theoretical contributions. First, this study investigates, for the first time, the important question of market concentration in the E-consultation context and compares it with the traditional offline health care market. The results indicate a superstar market rather than a long-tail market.

Second, previous studies on health care market concentration have mainly been conducted at the hospital level. Due to data limitations, very few studies have investigated the health care market concentration at the level of individual doctors. However, secondary data from an E-consultation website provided a unique opportunity to explore this important question at the individual doctor’s level.

Third, this study explores possible ways to decrease E-consultation market concentration from the information asymmetry perspective. Our findings reveal that two types of information disclosure mechanisms (ie, user feedback-based reputation and online self-representation) help to balance the supply and demand of health care service, which results in improved market efficiency.

### Limitations

This study has several limitations. First, only cross-sectional data were used in this study. Therefore, the role of intertemporal factors cannot be explored, and influences from many specific individual attributes cannot be completely eliminated. In the future, the panel data analysis method could be incorporated. Panel analysis uses panel data to examine changes in variables over time and differences in variables between subjects. The panel data contain rich information and would allow us to control for specific indicators. If the theory we proposed is correct and the data are sufficient, the results from panel analysis should be consistent with the cross-sectional analysis.

Second, data on only three disease types and from only one website (haodf.com) were used in this study. Therefore, the results of this study may not be fully representative of all diseases and the whole E-consultation market. In the future, we will continue this research by collecting data from multiple E-consultation websites and for more disease types.

### Conclusions

Our findings suggest that the E-consultation market is more concentrated than the offline market, exhibiting a superstar effect. Meanwhile, concentration can be reduced if the doctor’s signals of quality are sent properly. A market served by many doctors with strong reputations or high levels of self-representation will be less concentrated.

These findings provide significant insights for E-consultation website designers as well as for policy makers. This research reveals that user feedback and online representation are two important mechanisms that E-consultation websites should provide and encourage. A possible and important way to reduce the market concentration of E-consultation services is to accumulate enough highly rated and highly self-represented doctors.

We intend to explore how the level of market concentration varies based on different conditions in the future. For example, how does level of concentration vary based on specific type of online services (eg, diagnosis, monitoring, or intervention services)? How does level of concentration vary based on different condition types (eg, acute vs chronic, high mortality vs low mortality, rare vs common, urgency vs non-urgency)? How does level of concentration vary based on the distribution of offline medical resources? Answering these research questions may help us better understand the impact of internet on health consultation market concentration.

## Appendix

Multimedia Appendix 1 Empirical model.

Multimedia Appendix 2 Robustness check.
