# Supplementary material for: The Impact of the Internet on Health Consultation Market Concentration: An Econometric Analysis of Secondary Data
Source: J Med Internet Res. 2016 Oct 28;18(10):e276. doi: 10.2196/jmir.6423 (PMC5106558; doi:10.2196/jmir.6423)
Supplement: Multimedia Appendix 1 [file jmir_v18i10e276_app1.pdf]

Empirical Model

We fit the sales, sales rank, reputation and self-representation data to the following log-linear relationship.

$$\ln(Sales_j) = \beta_0 + \beta_1 \ln(Sales\ Rank_j) + \beta_2 Reputation_j + \beta_3 Self\text{-}Representation_j + \beta_4 \ln(Sales\ Rank_j) \times Reputation_j + \beta_5 \ln(Sales\ Rank_j) \times Self\text{-}Representation_j + \varepsilon_j \quad (1)$$

The β_1 coefficient measures how quickly doctor *j*'s demand falls as the sales rank increases, which indicates market concentration. If the E-consultation has a long tail effect, then β_1 will be less negative. In contrast, if the E-consultation has a super-star effect, then β_1 will be more negative.

The β_4 on the interaction term measures the moderating effect of online reputation on market concentration. If online reputation weakens market concentration, then β_4 should be positive and significant (given β_1 is negative and significant). In contrast, if online reputation strengthens market concentration, then β_4 should be negative and significant.

In the similar way, the β_5 on the interaction term measures the moderating effect of self-representation on market concentration. If self-representation weakens market concentration, then β_5 should be positive and significant (given β_1 is negative and significant). In contrast, if self-representation strengthens market concentration, then β_5 should be negative and significant.
