# Supplementary material for: The Impact of the Internet on Health Consultation Market Concentration: An Econometric Analysis of Secondary Data
Source: J Med Internet Res. 2016 Oct 28;18(10):e276. doi: 10.2196/jmir.6423 (PMC5106558; doi:10.2196/jmir.6423)
Supplement: Multimedia Appendix 2 [file jmir_v18i10e276_app2.pdf]

Robustness Check

We ran a robustness check by using alternative measures for reputation and self-endeavor (shown in Table 4). In the main analysis, reputation is measured by the combination of votes, gifts and thank-you letters. In the robustness check, reputation is measured by votes (Model 5), gifts (Model 6) or thank-you letters (Model 7), respectively. The model parameter estimates remain consistent with the main results in Model 3.

Similarly, in the main analysis, self-representation is measured by the combination of number of papers posted and number of free services provided. In the robustness check, we measure self-representation using number of papers (Model 9) or number of free services (Model 10), respectively. The model parameter estimates remain the same as the result in Model 4. Therefore, the results reported in the main analysis are robust.

Table 4. Robustness Check.

Variable	Model 5	Model 6	Model 7	Model 8	Model 9
Position	-0.007<br>(0.019)<br>*P*=.71	0.003<br>(0.019)<br>*P*=.87	-0.002<br>(0.019)<br>*P*=.91	-0.0003<br>(0.020)<br>*P*=.99	0.004t<br>(0.017)<br>P=.84
Level	0.015<br>(0.038)<br>*P*=.69	0.003<br>(0.039)<br>*P*=.93	0.011<br>(0.038)<br>*P*=.78	0.007<br>(0.040)<br>*P*=.86	0.024<br>(0.034)<br>*P*=.48
Price	0.0004<br>(0.0005)<br>*P*=.35	0.0001<br>(0.0005)<br>*P*=.85	0.0005<br>(0.0005)<br>*P*=.32	-0.0001<br>(0.0005)<br>*P*=.81	-0.001+<br>(0.0004)<br>*P*=.08
Duration	-0.0002<br>(0.001)<br>*P*=.84	0.0003<br>(0.001)<br>*P*=.72	-0.0001<br>(0.001)<br>*P*=.88	0.0001<br>(0.001)<br>*P*=.87	-0.001<br>(0.001)<br>*P*=.34
Lrank	-2.181***<br>(0.025)<br>*P*=.000	-2.131***<br>(0.023)<br>*P*=.000	-2.178***<br>(0.024)<br>*P*=.000	-2.048***<br>(0.023)<br>*P*=.000	-2.579***<br>(0.031)<br>*P*=.000
Reputation(vote)	-0.028***<br>(0.002)<br>*P*=.000				
Reputation (vote)*Lrank	0.006***<br>(0.0003)<br>*P*=.000				
Reputation (gift)		-0.013***<br>(0.001)<br>*P*=.000			

Reputation(gift)*Lrank		0.003***<br>(0.0002)<br>*P*=.000			
Reputation (thank-you letter)			-0.079***<br>(0.004)<br>*P*=.000		
Reputation (thank-you letter)*Lrank			0.016***<br>(0.001)<br>*P*=.000		
Self-Representation(articles)				-0.020***<br>(0.002)<br>*P*=.000	
Self-Representation (articles)*Lrank				0.004***<br>(0.0003)<br>*P*=.000	
Self-Representation (free service)					-0.001***<br>(0.000)<br>*P*=.000
Self-Representation (free service)*Lrank					0.0002***<br>(0.000)<br>*P*=.000
Constant	16.269***<br>(0.211)<br>*P*=.000	16.072***<br>(0.209)<br>*P*=.000	16.282***<br>(0.209)<br>*P*=.000	15.607***<br>(0.210)<br>*P*=.000	18.987***<br>(0.241)<br>*P*=.000
R2	0.812	0.811	0.815	0.798	0.85

Note: The number in parentheses are standard error. +*P*<.1; **P*<.05; ***P*<.01; ****P*<.001.
